# Experimental validation of the prestretch-strain relationship as a non-invasive index of left ventricular myocardial contractility

**DOI:** 10.1371/journal.pone.0228027

**Published:** 2020-02-26

**Authors:** Oana Mirea, Carolina Vallecilla, Piet Claus, Frank Rademakers, Jan D’hooge

**Affiliations:** 1 Department of Cardiovascular Imaging and Dynamics, University of Leuven (KU Leuven), Leuven, Belgium; 2 Department of Cardiology, University of Medicine and Pharmacy of Craiova, Craiova, Romania; Faculty of Medical Science - State University of Campinas, BRAZIL

## Abstract

**Background:**

The slope of the relationship between segmental PreS and total systolic shortening (S) has been proposed as a non-invasive index of left ventricular contractility. The aim of this study was to correlate this novel parameter to invasive gold standard measurements of contractility and to investigate how it is influenced by afterload.

**Methods:**

In domestic pigs, afterload was increased by either balloon inflation in the aorta or by administration of phenylephrine while contractility was increased by dobutamine infusion. During all interventions, left ventricular pressure-volume measurements and trans-diaphragmatic two-dimensional echocardiographic images were acquired. The PreS-S slope was constructed from 18 segmental strain curves obtained by speckle tracking analysis and compared to the slope of the end systolic PV relationship (E_max_) and the pre-load recruitable stroke work (PRSW).

**Results:**

Sixteen datasets of increased contractility and afterload were analyzed. During dobutamine infusion, the LV volumes decreased (p<0.05) while ejection fraction increased (p<0.05). Emax, PRSW and the slope of the intra-ventricular PreS-S relation increased significantly during dobutamine infusion. Afterload increase led to increase in systolic blood pressure (105±16mmHg vs. 138±25mmHg; p<0.01) and decrease of LV stroke volume and ejection fraction (p<0.01). The PreS-S slope was not influenced by loading conditions in concordance with the PRSW findings. The absolute values of the PreS-S slope did not correlate with Emax or PRSW. However, the change of the PreS-S slope in relation with different interventions demonstrated good correlation with changes in PRSW or Emax, (r = 0.66, p<0.05 and r = 0.69, p<0.05).

**Conclusions:**

The slope of the PreS-S relationship is sensitive to changes in inotropy and is less load-dependent than conventional non-invasive parameters of left ventricular function. The magnitude of the change of this slope correlates well with changes in invasive contractility measurements making it an attractive parameter to assess contractile reserve or contractile changes during longitudinal follow-up of patients.

## Introduction

Despite the remarkable progress in cardiovascular research, the accurate (non-invasive) quantification of left ventricular (LV) contractile function remains a major challenge in clinical practice. As such, many studies have focused on identifying the best possible index of LV contractility taking into account its load dependency, its sensitivity to inotropic and chronotropic state and its reproducibility [1–11].Currently, the slope (E_max_) of the end systolic pressure volume relationship (ESPVR) and the preload recruitable stroke work (PRSW) are considered the gold standards measurements of LV contractility. Additionally, the slope of the dP/dt_max_-end diastolic volume (EDV) relationship is also commonly used as an estimate of LV contractility. All these measurements are based on the Frank-Starling law of the heart that describes a direct relation between preload and the active force developed by the ventricle [1–5,8]. Available data from experimental studies on mammals and humans points towards PRSW as the most reliable index due to its load independency, its sensitivity to contractile changes and its low measurement variability [1,3,5,6,7,9]. In contrast, the maximal slope of the dP/dt_max_-EDV relationship was reported to be load dependent and sensitive to measurement variability while data on E_max_ is contradictory, with some studies reporting a degree of load dependency and non-linearity of the measurement under non-physiologic conditions while others do not [1–6,10–15]. Of course, the invasive nature by which these parameters are obtained is the most important limitation to its clinical routine use. Indeed, the acquisition protocol requires intra-cardiac pressure and volume measurements and can be difficult especially in heart failure or critically ill cardiac patients [2,16]. This, along with the complex, time-consuming post-processing analysis required, hampers the implementation of these indexes on a routine basis. The single beat derived measurements have been proposed as a noninvasive parameter for assessing the ventricular elastance [17]. This method employed measurement of systolic and diastolic arm-cuff pressures, echo-derived stroke volume and ejection fraction. However, this intricate multi-parametric approach limited the clinical implementation of the method.

Hence, there is a clear need for a non-invasive methodology for the assessment of LV contractility that can not only be easily implemented in clinical routine, but that also shows to be reproducible, load independent and sensitive to contractile changes.

In a previous study, we hypothesized that the slope of the intra-ventricular relationship between the regional myocardial stretch during atrial contraction, i.e. prestretch (PreS), and the associated regional systolic strain (S) could be an index of LV contractility [8]. The slope of the intra-ventricular PreS-S relationship was found to be relatively constant throughout all age groups, insensitive to changes in preload and sensitive to changes in the contractile state in a clinical setting and as reproducible as conventional measurements of myocardial function [8,18]. However, to date, a rigorous validation of its relationship to gold standard measurements of contractility is lacking nor is the effect of afterload on this novel index understood.

The purpose of this study was therefore two-fold: i) to contrast its sensitivity to changes in inotropy against gold standard methods of LV contractility and ii) to investigate the load dependency of the slope of the intra-ventricular PreS-S relationship.

## Materials and methods

### Experimental preparation

The experimental protocol was conducted in twenty-three domestic pigs of either gender (33.0 ±4.0 kg) in conformity to the National Institutes of Health Guide for the Care and Use of Laboratory Animals and was approved by the ethical committee of the Medical Faculty of the KU Leuven, Belgium.

### Medication

The animals were pre-medicated with an intramuscular mixture of zoletil (8mg/kg) and xylazine (2,5 mg/kg). Once positioned in dorsal recumbency and after endotracheal intubation, anesthesia was induced with an intravenous bolus of propofol (3mg/kg). The animals were connected to a ventilator and the initial tidal volume (9 ml/kg) was adjusted in each experiment to provide adequate arterial oxygen saturation. Continuous anesthesia was maintained with an intravenous mixture of propofol (10 mg/kg/h) and remifentanil (18 μg/kg/h). For prevention of thromboembolism and clotting of the vascular catheters, a bolus of heparin (10.000 IU) was administrated during instrumentation. After each experiment, animals were euthanized with an overdose of over-saturated potassium chloride solution under deep anesthesia.

### Instrumentation

An 8Fr sheath was inserted in the right carotid artery (RCA) and used for the introduction of the conductance tip catheter (Millar Instruments, Inc, Houston, TX) in the LV apex. A femoral artery and a femoral vein were accessed by deep inguinal puncture and a 9Fr introductory system sheath was placed in each access for the subsequent balloon catheter introduction. Under fluoroscopic guidance, the balloons were positioned in the inferior vena cava and the descending aorta, respectively.

Finally, a transversal superior laparotomy was performed parallel to the costal margins for acquisition of echocardiographic data.

### Interventions

In order to test the dependency of the PreS-S relationship on physiologic changes, the following interventions were performed. The interventions did not follow a strict order to avoid preconditioning bias.

Afterload increase with balloon inflation (IAB). The balloon placed in the descending aorta was gradually inflated until an increase of minimum 15% of the basal LV pressure was obtained. In three pigs, the afterload increase was repeated during pharmacologic attenuation of the autonomic reflexes, which was induced by infusion of atropine methyl nitrate (3 mg/kg), propranolol hydrochloride (2 mg/kg) and hexamethonium bromide (20 mg/kg). This was performed to add extra datasets in which the reflex changes in the contractile state during caval occlusion were prevented and the intrinsic heart rate (HR) of the animal was maintained.

Afterload increase with infusion of phenylephrine (IAP). In order to obtain a wide range of loading conditions, in a subgroup of animals, afterload was increased by administration of an α_1_-adrenergic agonist (phenylephrine, 5 μg/kg IV). In this scenario, the changes in afterload were the result of a more general vasoconstriction.

Changes in contractility (IC). Contractility was increased by a continuous infusion of dobutamine (6 μg/kg/min) for 10 minutes.

### Data collection

For all interventions, a set of echocardiographic images as well as PV loop data were acquired before, during and after the intervention. Before any data recording, the animal was given sufficient time to reach a stable condition.

### Two-dimensional echocardiography

Echocardiographic data sets were acquired using a trans-diaphragmatic approach using a GE E9 system (GE Vingmed Ultrasound, Horten, Norway) equipped with a 2.5 MHz M5S transducer. Prior to the acquisition, the ultrasound system settings were optimized in order to optimize image quality. The protocol included grey scale recordings of the 2, 3 and 4 chamber views at a minimum frame rate of 50fps and Pulsed wave (PW) Doppler recordings obtained by sampling the LV outflow tract. For each recording, at least 3 consecutive cardiac cycles were acquired and stored for off-line analysis.

### Pressure-volume loops

Prior to the insertion, the micromanometer was calibrated at 38°C and zeroed at mid-thorax level. The fall in LV pressures and volumes during preload reduction by means of transient caval occlusion as a result of inflation of the balloon in the IVC, was continuously recorded using a MPVS Ultra® Pressure-Volume Loop System (AD Instruments, Texas, USA). Immediately after the PV loop acquisition, the IVC balloon was deflated in order to limit the time duration of the reduced preload condition as much as possible.

## Data analysis

### Two-dimensional echocardiographic measurements

The digital cine loops were analyzed offline using EchoPac version BT13 (GE Vingmed Ultrasound, Horten, Norway). All measurements were performed by a single experienced reader (OM). Left ventricular end systolic volume (ESV) and end-diastolic volume (EDV) as well as LV ejection fraction (EF) were measured from the apical 2 and 4 chamber views using Simpson’s biplane method as recommended in the current guidelines [19]. Stroke volume (SV) was calculated as the difference between LV EDV and ESV.

### Two-dimensional speckle tracking measurements

Myocardial deformation was analyzed using a predefined 18 segments model of the LV (i.e. 6 segments for each of the 3 echocardiographic views). In contrast to what is typically done in strain echocardiography, the P-wave (i.e. the beginning of atrial contraction) rather than the onset of the QRS complex of the ECG was chosen as the beginning of the deformation cycle. As such, the underlying assumption was made that the ventricle remains in a condition of minimal stress during diastasis (i.e. its un-deformed state). The analysis was done by manually tracking the LV endocardial border and the automatically generated region of interest was adjusted as required. Tracking was visually verified and segments were excluded for further the analysis in case of poor tracking quality due to the presence of motion artifacts or reverberations.

### Measurement of PreS, total_S and the slope of the intra-ventricular PreS-S relationship

**Fig 1:**
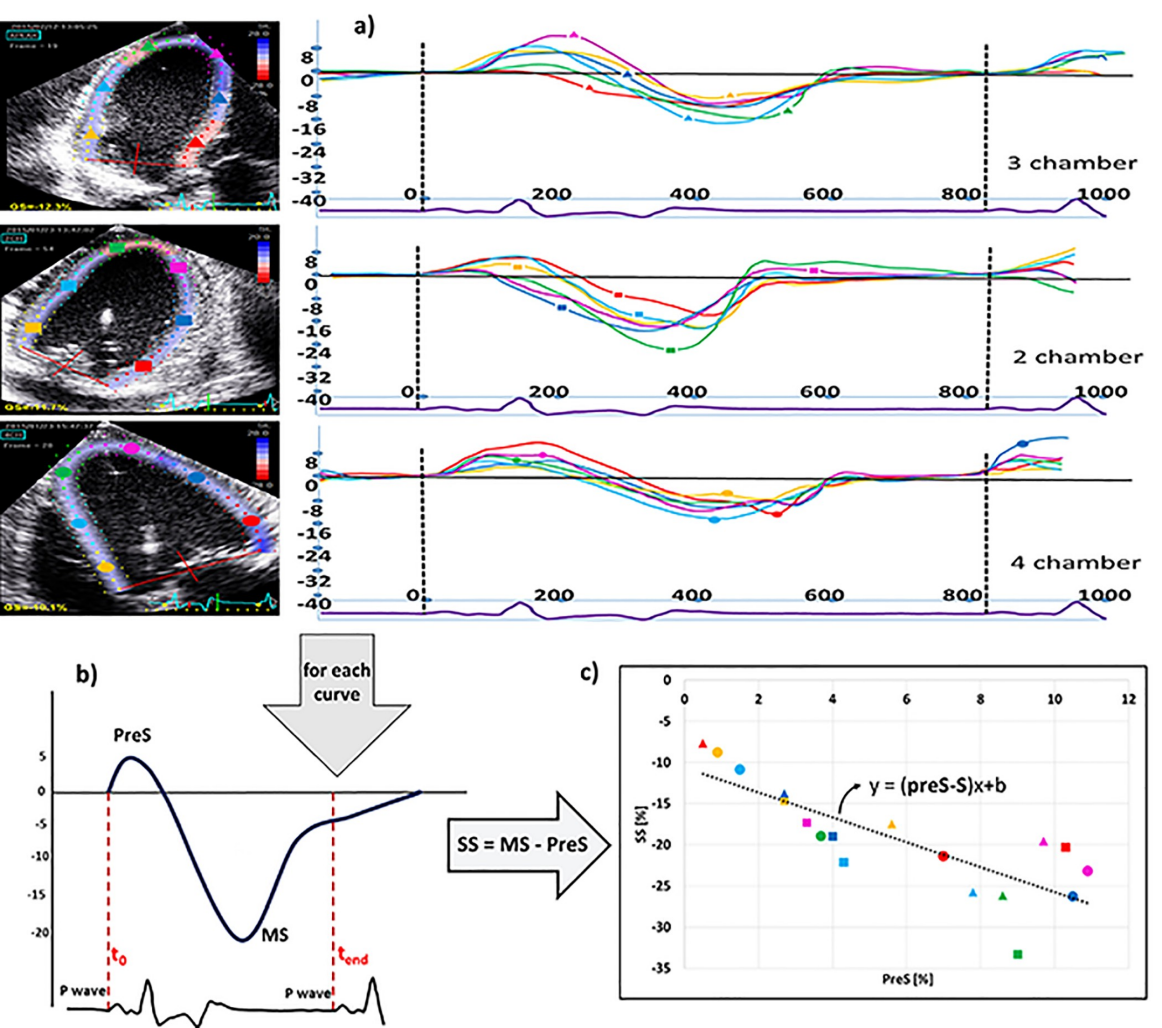
Measurement of Pre S, total_S and the slope of the intra-ventricular PreSS relationship. a) segmental myocardial deformation curves; b) definition of the total systolic shortening. PreS: peak positive strain (%),SS: total systolic shortening, MS: minimal strain near end-systole, c) PreS and SS values plot and the PreS-S slope.

From the obtained segmental myocardial deformation curves (Fig 1A), PreS was defined as the peak positive strain (%) while the associated total systolic shortening (S (%)) of that segment was defined as the difference between the peak positive strain and the minimal strain near end-systole (MS) as illustrated in Fig 1B. Subsequently, the 18 PreS and S values obtained within each ventricle were plotted in a 2D graph (Fig 1C) and the slope of their relationship determined by linear regression analysis. In case the Pearson’s correlation coefficient (R) of this regression was smaller than 0.6, the fit of the relationship was considered inadequate and the slope of the regression was omitted for further analysis.

### Pressure-volume loop analysis

All PV analyses were carried out using Labchart Pro (v7.3.7, AD Instruments, Sydney, Australia). In order to calibrate the volumes measured by the conductance catheter, ESV and EDV as measured by echocardiography (cf. above) for each animal and at each interventional state were used. E_max_ was measured by linear regression of the LV end-systolic pressure and volume changes during preload reduction. Similarly, PRSW was calculated by linear regression of the stroke work (i.e. the integral of a single-beat PV loop) and the EDV during IVC balloon inflation [5,7,15]. Finally, arterial elastance (E_a_) was estimated as (ESP-EDP)/SV [20]. If during the PV recordings an episode of tachycardia or extra systoles was present, data from those loops were not used for analysis.

### Statistical analysis

Clinical and echocardiographic characteristics are provided as mean ± SD for continuous variables, or as absolute number or percentage for categorical variables. Variables were checked to be normally distributed and to have equal variances. To compare data between the predefined study groups a paired t-test was used. Statistical analyses were performed using the software SPSS version 17.0 (SPSS Inc., Chicago, IL). Significance was set at a two-tailed probability level of p<0.05.

## Results

### Data analysis

The experimental protocol was successfully completed in 13 of the 23 animals. The exclusion of some of the data of 10 animals was done prospectively and due to both technical issues which hampered the interpretation and animal instability during the experiment. In detail, six animals were excluded due to: a) poor quality PV loops (n = 2), b) impossibility to acquire optimal echocardiographic recordings (n = 1), c) technical problems storing the data (n = 1), d) low correlation between the PreS and S values (n = 2), which was considered to be related to suboptimal image acquisitions since the correlation between PreS and S values was shown to be excellent. Additionally, four animals were excluded because of hemodynamic instability during the experiments due to arrhythmias or sudden death (n = 2) and suspicion of pericarditis (n = 2).

**Fig 2:**
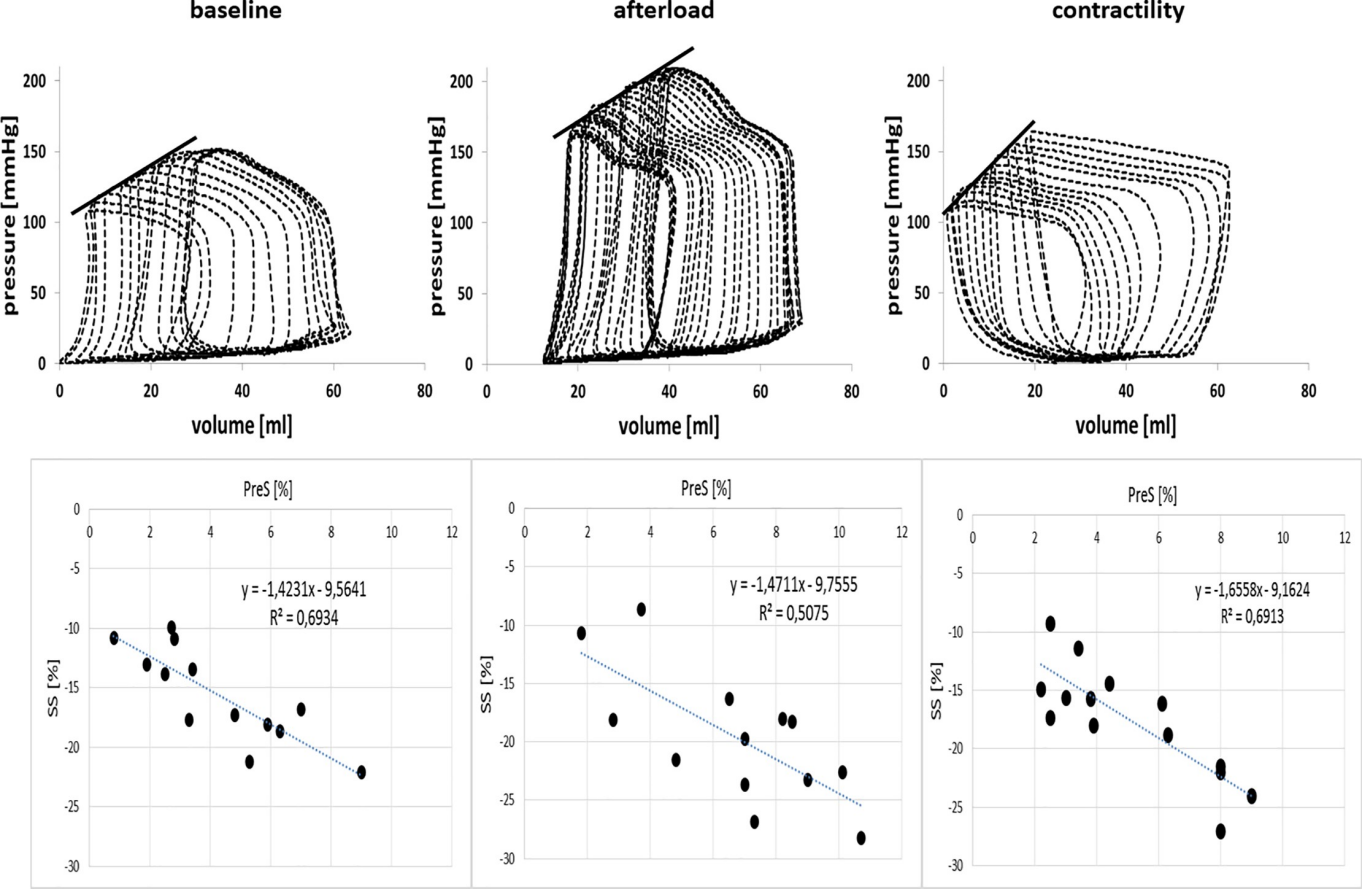
Representative intra-ventricular pressure-volume relations.

Table 1 details the available data. Averaged over all animals and interventions, 13±5 PV loops were recorded per preload reduction and thus used to extract the invasive measures of contractility and loading. The average correlation coefficients of the linear regressions performed were 0,98±0,01 for E_max_ and 0,96±0,03 for PRSW. Similarly, the average correlation coefficient obtained while fitting the intra-ventricular PreS-S relationship was 0.75±0.09. Representative intra-ventricular pressure-volume relations and the correlative PreS-S relationships are given in Fig 2.

**Table 1:** General description of interventions included in each subject.

Interventions per subject				
Subject	IAB	IAP	ANSB	IC
2	N	N	N	√
5	√	N	N	N
8	√	N	N	N
10	N	N	√	N
11	√	N	N	N
13	√	N	N	N
14	√	N	√	N
16	√	N	√	N
19	√	√	N	√
20	√	√	N	√
21	√	N	N	√
22	√	√	N	√
23	N	N	N	√

ANSB, autonomous nervous system blockade, IAB, increased afterload balloon; IAP, increased afterload phenylephrine; IC, increased contractility; √: performed and included; N, not performed

### Effect of changes in contractility

In response to the dobutamine infusion, systemic blood pressure did not change significantly while HR showed a marked increase. Compared to baseline values, the LV volumes decreased (p<0.05) while ejection fraction increased significantly (p<0.05). E_max_, PRSW and the slope of the intra-ventricular PreS-S relation increased significantly during dobutamine infusion. These results are presented in Table 2.

**Table 2:** Measurements during contractility increase.

Parameter	Unit	Baseline (n = 6)	IC (n = 6)
SBP	mmHg	99.7±16.0	116.7±24.4
HR	bpm	76.0±24.5	97.2±10.0*
EDV	ml	60.5±9.6	53.8±9.1*
ESV	ml	27.5±4.7	18.3±4.8*
EF	%	54.6±3.4	65.9±6.7*
SV	ml	33.0±5.7	35.5±7.1
PreSS	%/%	-1.66±0.46	-1.89±0.42
E_max_	mmHg/ml	0.84±0.37	1.81±1.13
PRSW	erg .10^5^/ml	0.43±0.28	0.81±0.45
Ea	mmHg/ml	-2.66±1.11	-3.35±1.49

ANSB, autonomic nervous system blockade; IAB, increased afterload balloon; IAP, increased afterload phenylephrine; IC, increased contractility; EDV, end diastolic volume; EF, ejection fraction; ESV, end systolic volume; SV, stroke volume; E_max_, slope of end systolic pressure volume relationship; PRSW, preload recruitable stroke work; Ea, elastance; SBP, systolic blood pressure; HR, heart rate.

*p<0.05 when compared to baseline.

**Fig 3:**
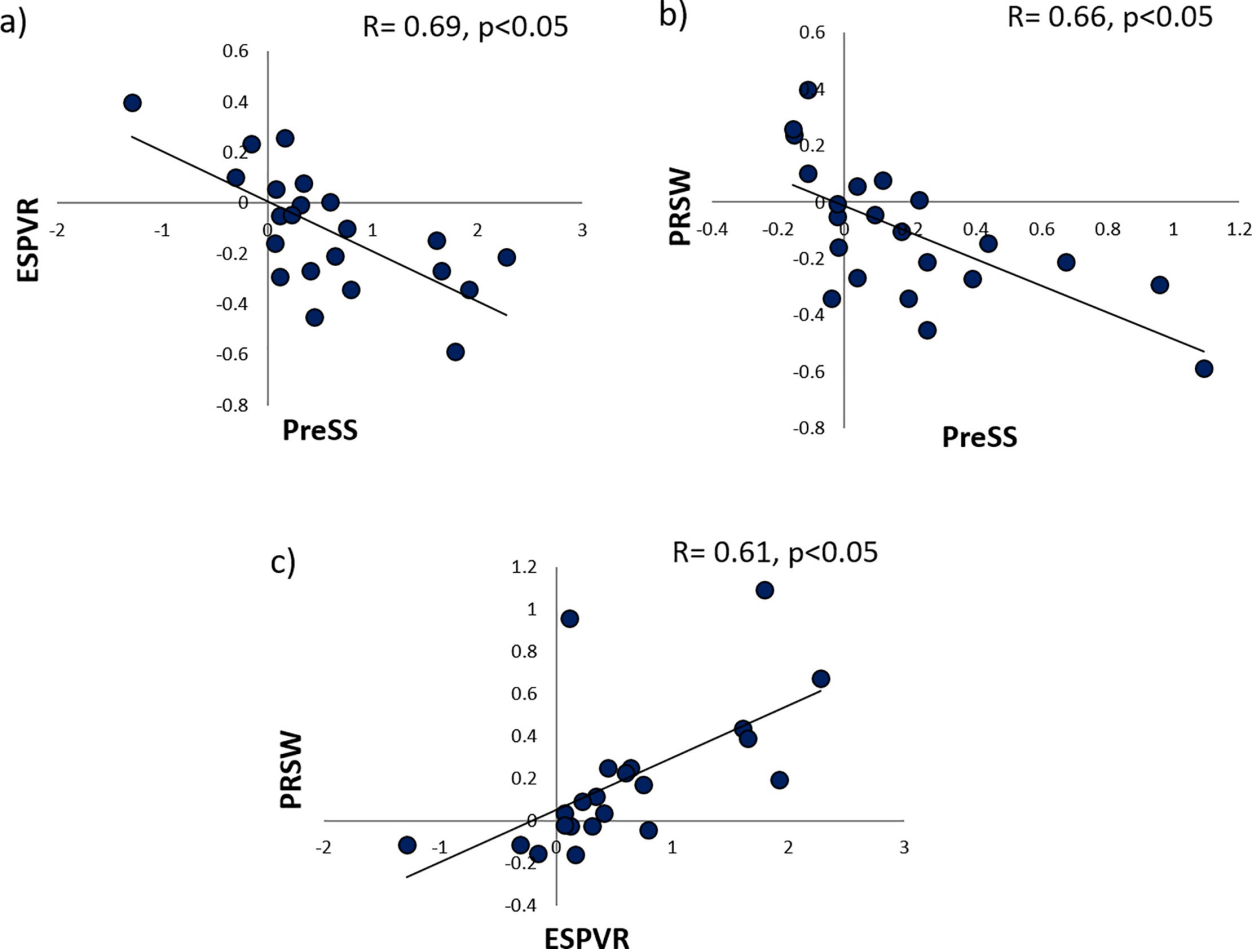
Correlations between changes in the slope of PreS-S slope and gold standard measurements of contractility. a) Correlation between changes in slope of PreS-S slope and E_max_. b) Correlation between changes in slope of PreS-S slope and PRSW c) Correlation between changes in slope of PRSW and E_max_.

No correlation was found between the absolute slopes of the intra-ventricular PreS-S relation and PRSW or ESPRV (p = NS). However, when correlating the changes in the slope of the intra-ventricular PreS-S relation with different interventions against the changes in E_max_ or PRSW during these interventions, correlation coefficients of 0.69 (p<0.05) and 0.66 (p<0.05) were found respectively (Fig 3A and 3B). Interestingly, the correlation between both gold standard methods was found to be in the same order of magnitude (r = 0.61, p<0.05) (Fig 3C).

### Effect of loading

General echocardiographic and hemodynamic measurements obtained under baseline conditions and during the changes in loading are listed in Table 3. During aortic balloon inflation, the blood pressure increased–on average–by 22.9±9.5% (p<0.01). In contrast, there was no significant change in heart rate. As expected, the afterload modulation triggered an increase in both end-diastolic and end systolic volumes (p<0.01) by 11.3±8.3% and 30.6±9.5% respectively. This was associated with a decrease in EF of 33.6±20.8% (p<0.01).

**Table 3:** Effect of afterload increase on steady state hemodynamic variables and echo measurements.

Parameter	Unit	Baseline (n = 16)	IA (n = 16)
SBP	mmHg	105±16	138±25*
HR	bpm	80±16	80±21
EDV	ml	64.00±8.50	72.38±8.99*
ESV	ml	28.6±6.36	41.31±6.80*
EF	%	54.76±7.73	41.65±7.35*
SV	ml	36.06±4.81	29.88±5.32*

IA, increased afterload; EDV, end diastolic volume; EF, ejection fraction; ESV, end systolic volume; HR, heart rate; SV, stroke volume; SPB, systolic blood pressure.

* p<0.01 when compared to baseline

The values for the calculated indexes of contractility are presented in Table 4. The slopes of the intra-ventricular PreS-S relationship and the PRSW showed no changes during altered loading conditions (p = NS). In contrast, E_max_ and Ea increased during increased loading (p<0.05). On average, SW did not change significantly during afterload increase.

**Table 4:** Effect of afterload increase on PreSS, E_max_, PRSW, SW and Ea.

Parameter	Unit	Baseline (n = 16)	IA (n = 16)
PreS-S	-	-1.39±0.22	-1.45±0.21
E_max_	mmHg/ml	1.40±0.74	1.88±0.72*
PRSW	erg .10^5^/ml	0.66±0.26	0.80±0.30
SW	erg .10^5^	44.59±9.87	50.71±12.34
Ea	mmHg/ml	-3.20±0.76	-5.04±1.49*

Ea, arterial elastance; E_max_, slope of end-systolic pressure volume relationship; PRSW, preload recruitable stroke work, SW, stroke work.

*p<0.05 when compared to baseline

## Discussion

The major findings of this study are that: i) the slope of the intra-ventricular PreS-S relation accurately estimates intra-animal changes in contractile state and ii) the slope of the intra-ventricular PreS-S relation is–in contrast to conventional non-invasive functional measures–not significantly influenced by loading conditions.

### Effect of changes in contractility

Administration of dobutamine decreased LV volumes and was associated with an increase in ejection fraction. These effects are well known and are due to incomplete filling as a consequence of uncompensated venous return in combination with high heart rate and increased inotropic state respectively [21]. Importantly, all contractile parameters tested (i.e. E_max_, PRSW and PreS-S) significantly increased in response to dobutamine (Table 2) demonstrating that the proposed index is indeed sensitive to the inotropic state of the myocardium. Moreover, changes of this parameter induced by interventions correlated well with the changes in inotropic state determined by gold standard measures demonstrating that intra-animal changes in contractility can correctly be monitored using this novel non-invasive parameter. Interestingly, this correlation was as strong as the correlation between both invasive gold standard measures of contractility further supporting the fact that the proposed parameter correctly represents contractile changes of the LV. Unfortunately, no correlation was found between the absolute value of the slope of the intra-ventricular PreS-S relationship and the invasive gold standards of contractility showing that absolute values of this parameter cannot be used as an absolute measure of contractility and that inter-animal differences in inotropic state cannot reliably be assessed by a mere description of this novel parameter.

### Effects of altered load

As expected, increasing afterload resulted in an increase in LV volumes and a decrease in both ejection fraction and stroke volume (Table 3). Given that invasive gold standard measurements of contractility did not show a decrease in contractility, this confirms the well-known fact that these conventional functional measures are not reliably representing contractile state in conditions of altered loading. In contrast, the slope of the intra-ventricular PreS-S relationship demonstrated no significant change during the afterload challenge suggesting an unaltered inotropic state of the myocardium. Although this was confirmed by PRSW measurements of ventricular contractility, this was not in agreement with the inotropic changes during loading as determined by E_max_ (cf. Table 4). However, previous studies have reported similar (conflicting) behavior of the invasive gold standard measures and have suggested that PRSW is a more robust parameter for measurement of the contractility [1,3–7]. This is consistent with our findings where the slope of the PRSW did not change with altered loading conditions.

In our study, loading was changed by aortic balloon inflation as well as by phenylephrine administration. The sole purpose of performing both interventions was to obtain a wider range of afterload challenges. We did thus not aim at exploring whether the two methodologies to alter loading had a different impact on left ventricular mechanics, which fell outside the scope of this validation study.

### Relationship with left atrial function

The concept of the index relies on the intrinsic variability in segmental LV curvature, which will result, according to Laplace (as a first approximation of wall stress)–in a segmental variation in wall stress during atrial contraction. As segmental strain is proportional to segmental stress this implies that strain during atrial filling (i.e. pre-stretch) is different for all LV segments. When LA functional changes would result in changes in LV pressure during atrial contraction, Laplace would indeed indicate that segmental stresses change accordingly and thus the induced strains (i.e. pre-stretch). Importantly however, the relative amount of pre-stretching of one segment with respect to another does not change as a result of such pressure changes if we assume we operate in the linear part of the myocardial stress-strain relationship; an assumption that seems reasonable given the relatively small pre-stretch resulting from atrial contraction (i.e. <10%). Indeed, increasing LV pressure will increase segmental stress (and thus pre-stretch) but the relative stress of one segment to another remains the same (and is determined by their relative curvature and wall thicknesses, i.e. morphology). Given that our method makes use of the slope of the prestretch-strain relationship, this slope will not be dependent on LA function as it is intrinsically accounted for. In fact, we believe this is one of the strengths of the proposed methodology.

### Limitations

The study protocol required the use of anesthesia which may, in theory, interfere with myocardial resistance and elastance. Nonetheless, our study was a validations study against gold standard measurements of contractility and the potential influence of anesthesia would have impacted both methodologies and thus not invalidate the study results.

Although in some animals the potential inotropic change due to acute afterload increase (i.e. as a consequence of the Anrep effect [22]) was avoided, this was not done in all animals. Unfortunately, the group of animals receiving ANS blockade was too small to draw conclusions and investigate this question. However, given that this study focused on validating a new non-invasive parameter to assess the contractile state of the LV by contrasting it over a range of physiologic states against invasive gold standard measures rather than to elucidate on the physiology of the acutely loaded heart, this was not considered an important limitation.

Several studies have questioned the performance of ESPVR given that the relationship is not always linear when experimenting in conscious mammals and have recommended other types of non-linear curve fitting in order to obtain a more accurate index of LV contractility [3,5,10–12,14]. However, in our study we found the relationship between end-systolic pressure and volume highly linear (cf. the high correlation coefficient found) and did not consider this a problem.

Preload is managed solely by reduction by vena caval balloon inflation. The preload increment procedures were not performed due to time constraints (the complexity of the study implied that all experiments required a prolonged period of time). On the other hand, in a previous clinical study of our group we could demonstrate that the index was not influenced by preload increase [8].

We recognize that dilated hearts will show limited myocardial pre-stretch as a result of atrial contraction similar to ventricles working on a steeper part of the pressure-volume relationship (i.e. less compliant ventricles). Although very small pre-stretch values may be difficult to measure reliably with the current echocardiographic methodologies, intrinsically the concept would hold as–even under these low average pre-stretch conditions–regional heterogeneity will remain resulting in heterogeneity in subsequent shortening thus allowing building a prestretch-strain relationship. Of course, the more globular shape of these dilated hearts will reduce the heterogeneity in regional curvature and therefore make the proposed methodology more difficult to apply.

The applicability of the proposed methodology in conditions of dilated or hypertrophied hearts or in the presence of regional myocardial ischemic abnormalities (ischemic heart disease) remains to be investigated in future clinical studies.

In the presence of atrial fibrillation or atrial flutter the pre-stretch cannot be reliably measured therefore the existence of these rhythm disturbances limits the use of the index.

Finally, the study was performed only in animals with normal resting diastolic function. Future investigations should be carried in order to study the value of the PreS-S slope in conditions of abnormal diastolic function.

### Clinical impact

The accurate quantification of LV function remains crucial in daily clinical practice. Despite the fact that several studies have proposed and investigated various indexes of myocardial contractility, EF is still the most commonly used index in clinical routine to assess the inotropic state of the heart. The obvious reasons for this are the fact that it can be measured non-invasively by multiple modalities in a relatively easy manner. However, it is well known that EF is load dependent (as demonstrated again in our study, cf. Table 3) and will provide an inaccurate assessment of LV function, particularly in patients with valvular heart disease or heart failure [9]. Other proposed non-invasive measurements such as fractional shortening, end-systolic volume or global longitudinal strain have been demonstrated to be also sensitive to loading [9, 23].

Our study demonstrates that the proposed non-invasive index is not load dependent and is sensitive to inotropic changes. Given that it can easily be measured bedside using speckle tracking echocardiography, this may bring an important new non-invasive parameter to clinical routine practice. However, further evaluation of this parameter in different clinical settings is needed.

## Conclusion

The slope of the intra-ventricular PreS-S relationship is sensitive to changes in inotropy and can be used to assess changes in contractile state non-invasively. Moreover, it was demonstrated that this parameter is load independent. It could be a useful tool for clinicians in the non-invasive assessment of changes in the contractile state of the LV.

## Supporting information

S1 Dataset LV_Contractility_Data. (XLSX)

## Abbreviations

ANS: Autonomic nervous system
SBP: Systolic blood pressure
HR: Heart rate
IAB: Increased afterload balloon
IAP: Increased afterload phenylephrine
IC: Increased contractility
LV: Left ventricle
EDV: End-diastolic volume
ESV: End-systolic volume
SV: Stroke volume
EF: Ejection fraction
